# A core epitope targeting antibody of SARS-CoV-2

**DOI:** 10.1093/procel/pwac042

**Published:** 2022-10-26

**Authors:** Simeng Zhao, Fengjiang Liu, Shizhen Qiu, Qiaoshuai Lan, Yiran Wu, Wei Xu, Junzi Ke, Jie Yang, Xiaoyan Liu, Kun Wang, Hangtian Guo, Shuai Xia, Fangfang Zhang, Jiabei Wang, Xiaowen Hu, Lu Lu, Shibo Jiang, Suwen Zhao, Lianxin Liu, Youhua Xie, Xiuna Yang, Haopeng Wang, Guisheng Zhong

**Affiliations:** iHuman Institute, ShanghaiTech University, Shanghai 201210, China; Shanghai Institute for Advanced Immunochemical Studies, ShanghaiTech University, Shanghai 201210, China; Innovative Center for Pathogen Research, Guangzhou Laboratory, Guangzhou 510005, China; School of Life Science and Technology, ShanghaiTech University, Shanghai 201210, China; Key Laboratory of Medical Molecular Virology (MOE/NHC/CAMS), School of Basic Medical Sciences and Biosafety Level 3 Laboratory, Fudan University, Shanghai 200032, China; iHuman Institute, ShanghaiTech University, Shanghai 201210, China; Key Laboratory of Medical Molecular Virology (MOE/NHC/CAMS), School of Basic Medical Sciences and Biosafety Level 3 Laboratory, Fudan University, Shanghai 200032, China; iHuman Institute, ShanghaiTech University, Shanghai 201210, China; School of Life Science and Technology, ShanghaiTech University, Shanghai 201210, China; iHuman Institute, ShanghaiTech University, Shanghai 201210, China; School of Life Science and Technology, ShanghaiTech University, Shanghai 201210, China; iHuman Institute, ShanghaiTech University, Shanghai 201210, China; School of Life Science and Technology, ShanghaiTech University, Shanghai 201210, China; Shanghai Institute for Advanced Immunochemical Studies, ShanghaiTech University, Shanghai 201210, China; Key Laboratory of Medical Molecular Virology (MOE/NHC/CAMS), School of Basic Medical Sciences and Biosafety Level 3 Laboratory, Fudan University, Shanghai 200032, China; iHuman Institute, ShanghaiTech University, Shanghai 201210, China; Department of Hepatobiliary Surgery, Anhui Province Key Laboratory of Hepatopancreatobiliary Surgery, The First Affiliated Hospital of USTC, Division of Life Sciences and Medicine, The University of Science and Technology of China, Hefei 230001, China; Department of Pulmonary and Critical Care Medicine, Division of Life Sciences and Medicine, The First Affiliated Hospital of USTC, The University of Science and Technology of China, Hefei 230026, China; Key Laboratory of Medical Molecular Virology (MOE/NHC/CAMS), School of Basic Medical Sciences and Biosafety Level 3 Laboratory, Fudan University, Shanghai 200032, China; Key Laboratory of Medical Molecular Virology (MOE/NHC/CAMS), School of Basic Medical Sciences and Biosafety Level 3 Laboratory, Fudan University, Shanghai 200032, China; iHuman Institute, ShanghaiTech University, Shanghai 201210, China; School of Life Science and Technology, ShanghaiTech University, Shanghai 201210, China; Division of Life Sciences and Medicine, The First Affiliated Hospital of USTC, The University of Sciences and Technology of China, Hefei 230001, China; Key Laboratory of Medical Molecular Virology (MOE/NHC/CAMS), School of Basic Medical Sciences and Biosafety Level 3 Laboratory, Fudan University, Shanghai 200032, China; Shanghai Institute for Advanced Immunochemical Studies, ShanghaiTech University, Shanghai 201210, China; School of Life Science and Technology, ShanghaiTech University, Shanghai 201210, China; iHuman Institute, ShanghaiTech University, Shanghai 201210, China; School of Life Science and Technology, ShanghaiTech University, Shanghai 201210, China

**Dear Editor,**

Tremendous efforts have been made globally to develop therapeutics and prophylactics against severe acute respiratory syndrome coronavirus-2 (SARS-CoV-2) which has caused thousands of millions of infections and deaths worldwide. A series of potent neutralizing antibodies with defined epitopes targeting RBD have been recently identified with different strategies. However, being an RNA virus, the instability of the SARS-CoV-2 genome results in numerous S-protein variants with altered viral phenotypes. For example, the dominant variant D614G shifts the S-protein conformation to a receptor-binding competent state with increased infectivity (Yurkovetskiy et al., 2020). Meanwhile, many naturally occurring mutant strains have been proven to be resistant to neutralizing antibodies or convalescent sera (Li et al., 2020). Recently, a newly emerged variant, B.1.351, which contains a E484K mutation, has been reported to escape neutralizing antibodies and first-generation vaccines (Chen et al., 2021; Wang et al., 2021). Though the cocktail strategies appear to be promising in the REGN-CoV-2 case, which was composed of two structurally well-defined antibodies targeting noncompeting epitopes (REGN10933 + REGN10987) (Baum et al., 2020; Hansen et al., 2020), novel escaping mutants may occur under selective pressure during long-term neutralizing antibody treatment. Indeed, a previous deep mutational scanning study showed that RBD is well tolerant to mutations (Starr et al., 2020), suggesting the need for the development of new antibodies to minimize viral escape.

To overcome this potential risk and understand the immune escaping mechanism of SARS-CoV-2 neutralizing antibodies, we performed comprehensive interface analyses of ACE2 receptor as well as structurally-resolved neutralizing antibodies. We grouped the antibodies into five classes based on their epitopes (Figs. S1 and S2): (i) Epitopes generally confined to the receptor-binding motif (RBM). (ii) Epitopes comprised of RBM and residues 403–421 and antibodies lean toward the buried side buried in the closed state (Wrapp et al., 2020). (iii) Epitopes primarily anchored to RBM and antibodies lean to the exposed face exposed in both the closed and open states (Wrapp et al., 2020). (iv) Epitopes located at the exposed side while antibodies minimally interact with RBM. (v) Epitopes located at the buried side while antibodies have almost no interactions with RBM.

We further validated the function of the structurally elucidated binding residues (Fig. S3A) of ACE2 receptor by calculating the binding affinities of mutated RBD proteins to ACE2 systematically. Mutations in residues F456 and N487 significantly weakened the binding affinity of ACE2 (Fig. S3B and S3D). Meanwhile, mutations in other residues spatially close to ACE2 (<4 Å) did not affect ACE2 binding (Fig. S3C and S3D), though they were thought to be responsible for receptor-binding (Lan et al., 2020). Newly emerged mutations, K417N, E484K, and N501Y, did not affect the binding of RBD to the ACE2 (Fig. S3B–D). Consistent with these results, few variants occurred in F456 and N487, while a series of mutations have been observed in other residues of RBM in natural strains (Fig. S3E). More surprisingly, we found that the core residues, F456 and N487, were also involved in the binding of Class I–II antibodies (Figs. S1 and S4A). We hypothetically defined this region as a “core epitope” and supposed that binding residue mutations outside of this core might result in an immune escape without compromising the infectious of viruses since these mutations dismissed the binding ability of antibodies but not ACE2. To support this idea, we chose REGN10933 (Class I) (Hansen et al., 2020), CB6 (Class II) (Shi et al., 2020) and BD368-2 (Class III) (Cao et al., 2020) as positive control antibodies and tested their affinity change by mutational scanning. Structure of REGN10933 in the complex of RBD revealed several binding residues outside of the core epitope (Fig. S4B) (Hansen et al., 2020). ELISA assay showed that mutations at Y453, L455, E484, and F486 weakened the binding ability of REGN10933 but not ACE2 (Fig. S4C–E), and mutants occurring in these residues have been reported to induce immune escape (Baum et al., 2020). Similar results were observed for CB6 and BD368-2 (Fig. S4C–E). These mutants did not affect the binding of ACE2 and consequently resulted in immune escape (Du et al., 2020; Li et al., 2020). We further tested the neutralizing activities of these antibodies against newly emerged variants, B.1.1.7 and B.1.351 (Chen et al., 2021; Wang et al., 2021). Results showed that B.1.351 variant, which contains K417N, E484K, and N501Y mutations, was immune escaping (Fig. S4F and S4G). Collectively, mutants outside of core residues were responsible for immune escape, which functionally support our “core epitope” hypothesis.

To further validate this hypothesis, we performed SARS-CoV-2 neutralizing antibodies screening experiment to discover core epitope targeting antibodies. We generated a phage-display library derived from PBMCs of seven COVID-19 recovered patients (Table S1). The capacity of individual primary libraries derived from each patient was between 8 × 10^6^ and 6 × 10^7^ (Fig. S5A). Sequence analyses indicated that more than 94.2% of variants in this library were effective (Fig. S5B). 3,840 single phage clones were picked randomly and analyzed by phage ELISA, and 420 clones exhibited positive ELISA signals and negative BSA signals (Fig. S5C). After sequencing 220 clones, we identified five re-emerged clones: 1F, 5E, 2B1, 2B8, and 2D1 (Fig. 1A; Table S4). These five antibodies were further tested for their RBD binding ability using ELISA. 1F exhibited the highest effectiveness, with EC_50_ value of 1.63 ng/mL (Fig. 1B). The *K*_D_ value was determined as 77 pmol/L using surface plasmon resonance (SPR) (Fig. 1C). We further tested the binding ability of 1F to mutated RBD proteins, and only the core residue mutation, N487A, could weaken the binding affinity of 1F (Fig. 1D–F). Mutations at other positions, including the new worrying mutation, E484K, did not affect the binding affinity (Fig. 1D–F). ACE2 competition using ELISA showed that 1F could block RBD-ACE2 binding efficiently, with an IC_50_ value of 0.69 μg/mL (Fig. 1G), consistent with the SPR result (Fig. 1H). Furthermore, 1F neutralized SARS-CoV-2 effeciently, with the IC_50_ values of 46.36 and 18 ng/mL against lentivirus-vectored SARS-CoV-2 pseudovirus and authentic virus, respectively (Fig. 1I and 1J). 1F also inhibited SARS-CoV-2 S-protein-mediated cell membrane fusion significantly at both 12 and 24 h, with IC_50_ values of 0.24 and 0.71 μg/mL, respectively (Figs. S6 and 1K). Finally, 1F possessed low neutralizing efficiency against SARS-CoV pseudovirus (Fig. 1L), indicating the specificity of the antibody.

**Figure 1.**
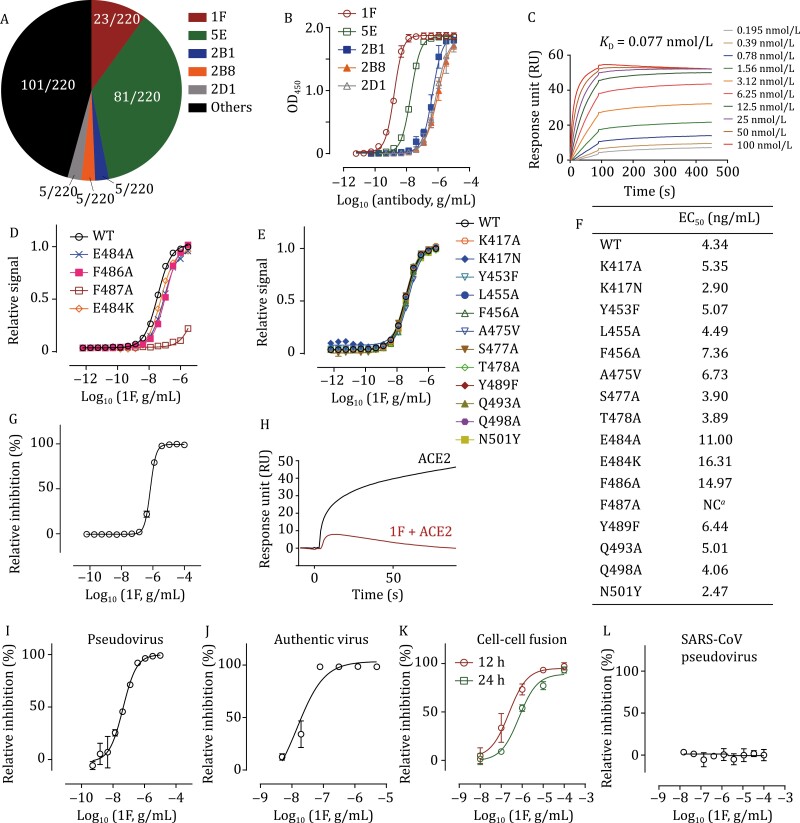
**Discovery and SARS-CoV-2 neutralization of 1F**. (A) Occurrence frequency of sequenced clones. (B) ELISA binding assay of selected antibodies to RBD protein. Experiments were performed in triplicate. (C) Representative SPR sensorgrams of 1F with RBD. (D and E) Binding ELISA assays of mutated RBD proteins on 1F. Experiments were performed in triplicate. (F) Summary of EC_50_ values in ELISA assays performed in (D) and (E). NC means EC_50_ cannot be calculated due to poor binding ability. (G) ACE2 competition of 1F by ELISA assay. Experiments were performed in quadruplicate. (H) ACE2 competition of 1F by SPR. Representative sensorgram of two independent experiments is shown. (I) Neutralization of 1F against lentivirus-vectored SARS-CoV-2 pseudovirus. (J) Neutralization of 1F on authentic SARS-CoV-2 virus. (K) Inhibition of 1F on SARS-CoV-2 spike mediated cell-cell fusion. (L) Neutralization of 1F against lentivirus-vectored SARS-CoV pseudovirus. All the experiments were performed in triplicates.

We then solved the cryo-EM structure of the 1F Fab in complex with the S-trimer. The SARS-CoV-2 spike ectodomain and 1F fab were expressed and purified (Fig. S7). After cryo-EM data collection and processing, we solved the cryo-EM structure of 1F Fab in complex with the S-trimer at an overall resolution of 3.8 Å (Figs. S8 and S9). A single 1F Fab binding to RBD at the open state was observed (Fig. 2A). Superimposition of the 1F–S complex structure over the ACE2-RBD complex structure revealed clashes between the ACE2 and 1F (Fig. 2B). To further improve the local resolution at the binding interface, we performed “block-based” local refinement and improved the local resolution up to 4.4 Å (Figs. S8 and S9D), allowing reliable tracing of the main chain at the interface. Only the heavy-chain of 1F was responsible for the antigen recognition and all the CDRs targeted the “core epitope” precisely (Fig. 2C). Since the resolution limited the analysis at the residue level, we predicted the spatially close residues (Fig. 2C) and performed mutation experiments to validate their role on antigen binding. Results showed that mutations at R102 or D114 (CDR3) dismissed the binding ability (Fig. 2D and 2E), indicating the critical role of these residues for RBD binding. We then tested the ability of 1F to avoid existing mutational immune escape (Baum et al., 2020; Du et al., 2020; Li et al., 2020). We first measured the neutralizing activities of 1F against the mutated pseudoviruses with previously reported escaping mutations (Li et al., 2020). Results showed that 1F could neutralize these escaping mutations effectively (Fig. 2F and 2H). The activities of 1F against several mutated pseudoviruses were significantly increased, such as V483A and F490L, which dismissed the effects of BD368-2 (Cao et al., 2020; Du et al., 2020). We further tested the effect of 1F against several other naturally occurred mutations. As expected, 1F neutralized these mutated pseudoviruses efficiently (Fig. 2G and 2H), including the dominant variant D614G (Yurkovetskiy et al., 2020). We then tested the neutralizing activity of 1F against newly emerging variants, B.1.1.7, B.1.351, and related RBD mutants. 1F inhibited B.1.1.7 efficiently (Fig. 2I and 2J). However, the neutralizing abilities of 1F against E484K and B.1.351 were significantly reduced (Fig. 2I and 2J). Since the binding of 1F to RBD was not affected by E484K mutation (Fig. 1D and 1F), we then performed ACE2 binding competition assay, and results showed that the E484K affected the dose-response curve slopes (Hill coefficient, 0.57 vs. 2.38) rather than the competing IC_50_ value of 1F (Fig. S10). These results indicated an underlying escaping mechanism that E484K mutation changed the inhibition slope of neutralizing antibodies.

**Figure 2.**
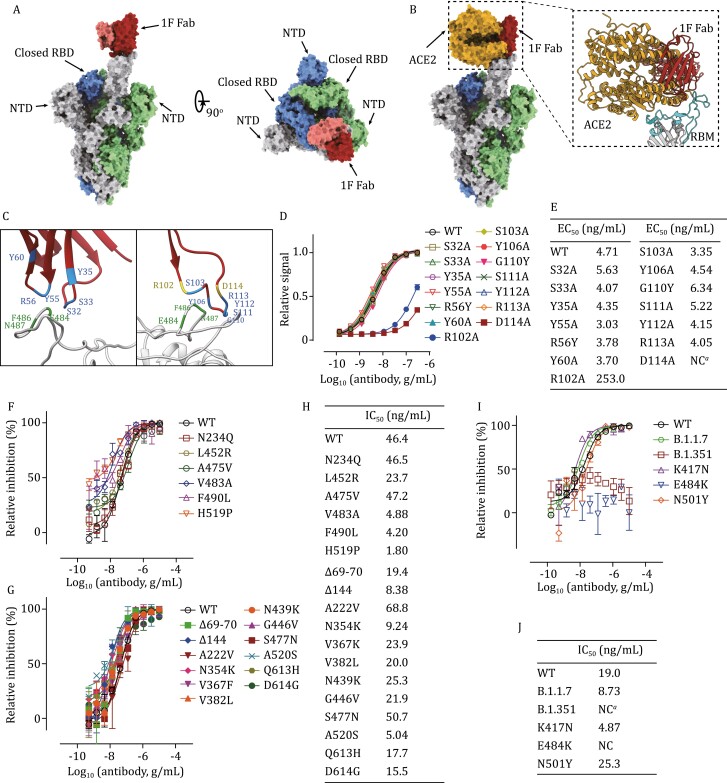
**1F binds to the core epitope and neutralize most of SARS-CoV-2 mutations**. (A) Overall structure of 1F Fab in complex with S-trimer. The structure is shown as a molecular surface with a different color for each S monomer as labeled, and the 1F Fab in red and pink (heavy-chain) and (light chain). (B) Clashes between 1F Fab and ACE2 on binding to SARS-CoV-2 S-trimer. ACE2 is shown in yellow. Inset shows close up of the interactions of 1F Fab and ACE2 in the clashed region, showing RBM in cyan. (C) The binding interface between 1F and S-protein, the spatially close residues were colored as indicated. (D) Binding ELISA assays of mutated 1F on RBD. Experiments were performed in triplicate. (E) Summary of EC_50_ values in ELISA assays performed in (D). NC means EC_50_ cannot be calculated due to poor binding ability. (F and G) The inhibitory dose curve of 1F on reported immune escape mutants (F) and naturally occurred mutants (G). (H) Summary of IC_50_ values of 1F in (F) and (G). (I) The inhibitory dose curve of 1F on recently emerged variants and related mutants. (J) Summary of IC_50_ values of 1F in (I), NC means IC_50_ cannot be calculated due to poor activity. Experiments were performed in triplicates.

Since reported, vast numbers of SARS-CoV-2 variants have occurred and some of them resulted in antigenic drift and immune escape (Li et al., 2020). Scientists have developed antibody cocktails to overcome this risk (Baum et al., 2020; Du et al., 2020; Hansen et al., 2020; Tortorici et al., 2020). However, RBD is well tolerated to mutations with respect to ACE2 binding as well as protein folding (Starr et al., 2020), and new escaping mutants may still occur under the selective pressure during long-term treatment. Here, by comprehensive structural analyses and extensive functional validation, we defined a core epitope avoiding viral escape. On the basis of this concept, we further identified an ultrapotent core epitope targeting antibody, 1F, which precisely binds to the core epitope and is tolerated to most of circulating mutations. However, the worrying mutant, E484K, which reduces the efficiency of neutralizing antibodies and first-generation vaccines (Chen et al., 2021; Wang et al., 2021), dismisses the neutralizing activity of 1F. Our data show that the reduced neutralizing activity of 1F against E484K result from reduced dose-response curve slope (Hill coefficient) (Fig. S10) rather than loss of binding affinity (Fig. 1D and 1F). A similar escaping mechanism has been observed in HIV (Sampah et al., 2011; Webb et al., 2015). Our results suggest distinct escaping mechanisms of E484 mutated SARS-CoV-2 variants.

## Supplementary Material

pwac042_suppl_Supplementary_Materials

## Footnotes

We thank the staff from the mammalian core, protein purification core, assay core, and molecular clone core of iHuman Institute, ShanghaiTech University for their assistance during protein expression and purification. We also thank the staff from the Bio-Electron Microscopy Facility of ShanghaiTech Univeristy for their assistance during cryo-EM data collection. We are grateful to the members of the Core Facility of Microbiology and Parasitology (SHMC) and the Biosafety Level 3 Laboratory at Shanghai Medical College of Fudan University, especially Qian Wang, Chengjian Gu, Xia Cai, Zhiping Sun and Di Qu. We are also grateful to Lei Sun from Fudan University for the kind gift of the construct to express the SARS-CoV-2 spike ectodomian.

This work was supported by ShanghaiTech, Shanghai Frontiers Science Center for Biomacromolecules and Precision Medicine at ShanghaiTech University (G.Z.), and grants from the National Key Research and Development Program of China [Grant Nos. 2017YFC1001301 (G.Z.), 2019YFA0111001 (H.W.)], the National Natural Science Foundation of China [Nos. 31670919 (H.W.), (81822045 (L.L.)], Lingang Laboratory (No. LG202101-01-07 to X.Y.), Program of Shanghai Academic/Technology Research Leader [20XD1420300 (L.L.)], Emergency response to new coronavirus infection scientific and technological project [(YD9110002004 (J.W.)], and Shanghai Sailing Program [20YF1430900 (S.-M.Z.)].

A patent based on this manuscript has been submitted. The patent applicant is ShanghaiTech University. The inventors are, Guisheng Zhong, Haopeng Wang, Simeng Zhao and Shizhen Qiu. Fengjiang Liu, Qiaoshuai Lan, Yiran Wu, Wei Xu, Junzi Ke, Jie Yang, Xiaoyan Liu, Kun Wang, Hangtian Guo, Shuai Xia, Fangfang Zhang, Jiabei Wang, Xiaowen Hu, Lu Lu, Shibo Jiang, Suwen Zhao, Lianxin Liu, Youhua Xie, and Xiuna Yang declare no competing interests.

This study received approval from the Research Ethics Committee of the First Affiliated Hospital of USTC, China. The research was conducted strictly in accordance with the rules of the Chinese government for the protection of human subjects. This study subjects signed the written informed consent for the use of their blood samples for research purposes.

Data generated and/or analyzed in the study are available on reasonable request from the corresponding author.

G.Z., H.W., and S.-M.Z. conceived and designed the experiments. S.-M.Z., J.K., J.Y., X.L. and F.Z. made all constructs, purified the proteins, and performed ELISA, SPR and pseudovirus neutralization assays. S.Q., K.W. and H.W. built phage-display library and performed antibody screening. F.L., H.G., and X.Y. collected and processed cryo-EM data and built and refined the structure models. Y.W. and S.-W.Z. performed structure analysis. Q.L., W.X., S.X., L.L., S.J., Y.X. performed authentic virus neutralization assay. J.W., X.H., and L.-X.L. collected the blood from COVID-19 convalescent patients. All authors contributed to data analysis, interpretation, and presentation. S.-M.Z. and G.Z. wrote the manuscript with contributions from all authors.
