# Supplementary material for: A core epitope targeting antibody of SARS-CoV-2
Source: Protein Cell. 2022 Oct 26;14(1):74–8. doi: 10.1093/procel/pwac042 (PMC9871958; doi:10.1093/procel/pwac042)
Supplement: pwac042_suppl_Supplementary_Materials [file pwac042_suppl_supplementary_materials.pdf]

Supplementary Information for

A core epitope targeting antibody of SARS-CoV-2

Authors: Simeng Zhao[1,9], Fengjiang Liu[2,3,9], Shizhen Qiu[4,9], Qiaoshuai Lan[5,9], Yiran Wu[1,9], Wei Xu[5,9], Junzi Ke[1,4,9], Jie Yang[1,4], Xiaoyan Liu[1], Kun Wang[4], Hangtian Guo[2], Shuai Xia[5], Fangfang Zhang[1], Jiabei Wang[6], Xiaowen Hu[7], Lu Lu[5], Shibo Jiang[5], Suwen Zhao[1,4], Lianxin Liu[8,*], Youhua Xie[5,*], Xiuna Yang[2,*], Haopeng Wang[4,*], Guisheng Zhong[1,4,*]

1. iHuman Institute, ShanghaiTech University, Shanghai 201210, China.

2. Shanghai Institute for Advanced Immunochemical Studies, ShanghaiTech University, Shanghai 201210, China

3. Innovative Center for Pathogen Research, Guangzhou Laboratory, Guangzhou, 510005, China

4. School of Life Science and Technology, ShanghaiTech University, Shanghai 201210, China.

5. Key Laboratory of Medical Molecular Virology (MOE/NHC/CAMS), School of Basic Medical Sciences and Biosafety Level 3 Laboratory, Fudan University, Shanghai 200032, China

6. Department of Hepatobiliary Surgery, Anhui Province Key Laboratory of Hepatopancreatobiliary Surgery. The First Affiliated Hospital of USTC, Division of Life Sciences and Medicine, The University of Science and Technology of China, Hefei, Anhui 230001, China.

7. Department of Pulmonary and Critical Care Medicine. The First Affiliated Hospital of USTC, Division of Life Sciences and Medicine, The University of Science and Technology of China, Hefei, Anhui 230026, China.

8. The First Affiliated Hospital of USTC, Division of Life Sciences and Medicine, The University of Sciences and Technology of China, Hefei, Anhui 230001, China.

9. These authors contributed equally.

Contact information

* liulx@ustc.edu.cn (Lianxin Liu)

*yhxie@fudan.edu.cn (Youhua Xie)

*yangxn@shanghaitech.edu.cn (Xiuna Yang)

*wanghp@shanghaitech.edu.cn (Haopeng Wang)

*zhongsh@shanghaitech.edu.cn (Guisheng Zhong)

Methods

Cells and viruses

HEK293T cells, eGFP expressing HEK293T (293T-eGFP), ACE2 expressing HEK293T (293T-ACE2), and Vero-E6 cells were maintained in DMEM medium supplemented with 10% FBS and penicillin/streptomycin under a humidified atmosphere of 5% CO_2 at 37 ^oC. 293T-eGFP and 293T-ACE2 cells were generated by lentivirus-mediated stable transduction. Briefly, 293T cells were co-transfected with FUW-eGFP or FUW-ACE2-P2A-PuroR and viral packaging plasmids, pCMVdR8.92 and pCMV-VSV-G. 72 h post transfection, the supernatant-containing virus was harvested and concentrated by ultracentrifugation. 293T cells were then infected with lentivirus to generate stably transfected cells. EGFP positive cells were sorted by FACS (BD Influx) and used as 293T-EGFP cells. 293T-ACE2 cells were selected using puromycin (Meilunbio) at a concentration of 2 μg/mL.

The authentic virus (SARS-CoV-2/SH01/human/2020/CHN, genbank: MT121215) was isolated from a COVID-19 patient in Shanghai. Viruses were amplified using Vero-E6 cells and stored at -80 ^oC. All experiments related to authentic virus were performed in a ABSL-3 laboratory at the Fudan University, Shanghai.

Protein expression and purification

For RBD and related mutant protein expression, coding sequences were cloned into a pTT5 vector with an N-terminal IL-2 signal peptide and a C-terminal 6 × His tag. The plasmids were transiently transfected into HEK Expi293F cells (Thermo Fisher Scientific) using polyethyleneimine (PEI). For SARS-CoV-2 S-trimer expression, the S-trimer (residues 1-1208) encoded DNA fragment was cloned into pTT5 vector with an N-terminal IL-2 signal peptide and a C-terminal 6 × His tag. Four substitutions at the cleavage site (residues 682-685) were changed to “GSAS” and two proline substitutions were introduced at the residues 986-987 to stabilize the protein expression(Wrapp et al., 2020). A T4 fibritin motif was engineered to stabilize trimerization. After a 5-day culture, supernatants were collected and proteins were purified with Ni-NTA resin (Qiagen) and Superose 6 10/300 gel filtration column (GE Healthcare) or Superdex 200 10/300 Increase column (GE Healthcare). Protein

concentrations were determined by BCA protein concentration kit (Pierce).

Antibody expression and Fab purification

Genes encoding heavy and light chains of antibodies were cloned into expression vectors with human IgG1 constant regions. Plasmids were transfected into HEK Expi 293F cells using PEI. Supernatants were collected after a 5-day culture, and antibodies were captured by protein A Sepharose (GE Healthcare). After washing with PBS, antibodies were eluted with 1M glycine (ABCONE). To obtain 1F-Fab, 1F antibody at a concentration of 2 mg/mL was digested with papain (0.1 mg/mL) in 50 mM phosphate buffer saline, pH 7.0, and 5.5 mM cysteine (SinoPharm) at 37 ^{o}C for 2 h. The reaction was terminated by adding iodoacetamide (Sigma). The Fc region was captured by protein A chromatography, and the Fab was then purified with the Superdex 200 10/300 Increase column. Concentrations of antibodies were determined using the BCA protein concentration kit.

ELISA

To evaluate the effects of RBD mutations on ACE2 receptor binding, Nunc Maxisorp plates (Thermo Fisher Scientific) were coated with purified wild-type or mutated RBD proteins at 2 μg/mL in PBS at 4 ^{o}C overnight. The plates were blocked using 5% skim milk at RT for 2 h after extensive washing with PBS. Biotinylated ACE2 (Novoprotein) was serially diluted and then added to the plate at RT for 1 h. After washing with PBS, HRP conjugated streptavidin (Proteintech) was added and incubated for 1 h. TMB substrate (Beyotime) was added after washing with PBS, and the absorbance at 450 nm was measured by a microplate reader (Flexstation III).

To test the binding affinity of antibodies to S-protein, RBD, and related mutant proteins, Nunc Maxisorp plates were coated with recombinant proteins at 2 μg/mL. After washing and blocking, serially diluted antibodies were then added to plates and incubated at RT for 1 h. After standard washing, HRP-conjugated goat anti-human IgG antibodies (Proteintech) were added and incubated for 1 h. After washing with PBS, the TMB substrate was added, and the absorbance was measured.

To perform the ACE2 competition assay, Nunc Maxisorp plates were coated with recombinant ACE2 (Novoprotein) at 2 μg/mL. After washing and blocking, 1F

antibody was serially diluted in 1 μg/mL of biotinylated RBD (Novoprotein) and added to plates at RT for 1 h. After standard washing, HRP conjugated streptavidin was used to detect the binding RBD. The absorbance was measured after adding the TMB substrate.

Phage display library generation

Peripheral blood mononuclear cells (PBMCs) were isolated from 10 ml of fresh blood from 7 different convalescent COVID-19 patients (Table S1). The fresh blood was density-gradient centrifugated on Histopaque-1077 (Sigma-Aldrich) following the specification. RNA was then extracted from the PBMCs using TRIZol Reagent (Invitrogen). cDNA was prepared from 8 μg of total RNA from each donor using PrimeScript II High Fidelity RT-PCR Kit (Takara), following the manufacturer's protocol. The immunoglobulin V_H and V_L genes were then amplified by PCR using 200 ng of cDNA as templates with specific primers as shown in Table S2. PCR products were then purified by gel extraction using NucleoSpin Gel and PCR Clean-up kit (Macherey-Nagel). About 200 ng of PCR product from each donor was used as a template for the subsequent PCR to introduce flanking homologous arms for Gibson assembly using the primers shown in Table S3. Products were purified and cloned into a phagemid vector HP153 (a gift from Shanghai Institute for Advanced Immunochemical Studies, ShanghaiTech University, Shanghai). The V_H PCR products were cloned into HP153 using Gibson assembly cloning kit (NEB). Recombination products were electro-transformed into *E. coli* JM109 cells (Takara) to generate V_H sublibraries. Phagemid DNAs from 7 different V_H sublibraries were extracted and restrictly digested with XhoI and NsiI, V_L PCR products were then cloned into V_H sublibraries. Ligated products were electro-transformed into *E. coli* SS320 cells (Lucigen) to generate the intact libraries.

RBD binder panning and Phage ELISA

Solid phase panning was used to select the specific binder of RBD as described (Tonikian et al., 2007). Purified RBD protein was coated on 96-well DNA-bind assay plates (Thermo Fisher Scientific) at a concentration of 10 μg/ml in PBS at 4℃ overnight. The plates were blocked with 2% BSA at room temperature (RT) for 1 h

and then used for RBD binder selection. BSA-coated plates were used as negative controls for nonspecific binder. Phages were concentrated by PEG-8000 and dissolved in PBS. After positive selection panning at RT for 1 h, the specific binders of BRD were eluted by benefit acidic solution and then amplified by infecting the *E. coli* TG1 (Beyotime). After two rounds of panning, single bacterial colonies were picked for further ELISA test. 96-well DNA-bind assay plates were coated with RBD protein at a concentration of 2 μg/ml at 4 ℃ overnight. After blocking with 2% BSA at RT for 1 h, the supernatant of single clone bacteria culture medium containing monogenotype phage were added to the plates and incubated at RT for 1 h. After extensive washing with PBS, HRP conjugated anti-M13 antibody (Sino Biological) was added and incubated for 1 h. The plates were further washed using PBS, and TMB substrate was added. The absorbance at 450 nm was then measured. Clones specifically bound to the RBD were sanger sequenced.

SPR analysis

The binding kinetics of 1F was analyzed using SPR (Biacore T200, GE Healthcare). Recombinant RBD was immobilized to a CM5 sensor chip in 10 mM sodium acetate buffer (pH 5.0) for final response units (RU) of about 100. SPR assays were run in PBST buffer (PBS with 0.005% Tween 20) at a flow rate of 30 μL/min. The sensograms were fit to a 1:1 binding model using Biacore Evaluation Software (GE Healthcare).

For the ACE2 competition assay, 1F (1 μM) were injected onto the chip for 90 s to reach steady binding state. ACE2 was then injected for 60 s to detect the blocking effect.

Cell-cell fusion assay

Cell-cell fusion assay was performed as described previously(Xia et al., 2020) with minor modifications. 293T-EGFP cells were transfected with pcDNA3.1-SARS-CoV-2-S plasmids using Lipofectamine 2000 (Invitrogen) to generate effector cells. 293T-ACE2 cells were used as target cells. To test the inhibitory activity of antibodies on SARS-CoV-2 mediated cell-cell fusion, effector cells and target cells were co-cultured in DMEM supplemented with 10% FBS and

serially diluted antibody. After incubation for indicated times, three to five fields were randomly selected and imaged using a fluorescence microscope (Nikon), and the numbers of fused and unfused cells were counted.

Pseudovirus neutralization assay

A pseudovirus neutralization assay was performed as previously described (Xia et al., 2020). Briefly, HEK-293T cells were co-transfected with pcDNA3.1-SARS-CoV-2-S or related mutants and pNL4-3.luc.RE to produce related SARS-CoV-2 pseudovirus particles. After a 72-h culture, the pseudovirus supernatant was collected and centrifuged at 3,000 X *g* for 10 min to remove dead cells. To test the neutralizing activity, 293T-ACE2 cells were seeded onto 96-well culture plates at a density of 5,000 cells per well. Antibodies were serially diluted with pseudovirus supernatant and incubated at 37 ^{o}C for 1 h followed by transferring to the target cells. After 12 h incubation, fresh cell culture medium was changed, and cells were further cultured for 48 h. Luciferase activities were then analyzed using luciferase assay substrate kit (Promega).

Authentic SARS-CoV-2 neutralization assay

Vero-E6 cells were seeded onto 96 well plates at the concentration of 10,000 cells per well. Serially diluted antibodies were added to the same volume of 100 PFU of SARS-CoV-2 and incubated for 1 h at 37 ^{o}C. The mixture was then added to the cells and incubated at 37 ^{o}C for 1 h. The culture medium was then transferred to DMEM medium supplemented with 2% FBS. After 48 h culture, the supernatant was collected, and the viral genome copies were determined using quantitative real-time PCR (qPCR). Viral RNA was extracted using TRIzol LS (Invitrogen). qPCR was then performed using One-Step PrimeScrip RT-PCR Kit (Takara) following the manufacturer’s protocol with primers as follows, SARS-CoV-2-N-F: GGGGAACTTCTCCTGCTAGAAT, SARS-CoV-2-N-R: CAGACATTTTGCTCTCAAGCTG, and SARS-CoV-2-N-probe: 5'-FAM-TTGCTGCTGCTTGACAGATT-TAMRA-3'.

Electron microscopy sample preparation and data collection

3 μL of purified SARS-CoV-2 S at 1.5 mg/ml was mixed with 1 μL of 1F Fab

fragments at 1.5 mg/ml in PBS buffer solution and incubated for 30 min on ice. A 3 μL aliquot of the mixture added with 0.025% DDM was transferred onto a freshly glow-discharged holey carbon grid (Quantifoil Cu R1.2/1.3). Grids were blotted for 2.5 s and flash-frozen in liquid ethane cooled by liquid nitrogen using a FEI Mark IV Vitrobot (FEI) at 8 ℃ and 100% humidity. Cryo-EM data were collected on a FEI Titan Krios electron microscope operated at 300 keV with a Gatan K3 camera at 29,000× nominal magnification in super-resolution mode and binned to a pixel size of 0.82 Å/pixel. All data were collected with a defocus range from -1.0 μm to -2.5 μm. Automated single-particle data acquisition was performed with SerialEM (Mastronarde, 2005).

Electron microscopy data analysis

Alignment and motion correction were performed using MotionCorr2(Zheng et al., 2017), with a five-by-four patch-based alignment. Subsequent steps were performed using cryoSPARC (Punjani et al., 2017). A total of 5,454 micrographs were recorded for the SARS-CoV-2 S 1F-Fab complex and aligned. Micrographs that exhibited defects in the Thon rings due to excessive drift, ice contamination, or astigmatism were discarded. Particles (989,274) were automatically selected, extracted from the micrographs with a box size of 448 binned to 224 and submitted for several rounds of reference-free 2D-classification to discard bad particles. After particle cleaning, 94,408 were used for Ab-Initio reconstruction to generate 3D models as references to perform heterogeneous refinement. After heterogeneous refinement, the S-trimer 1F-Fab complex class with 61,103 particles were re-extracted from micrographs with a box size of 448 and then refined using NU-Refinement to generate the final cryo-EM map with an estimated average resolution of 3.8 Å according to the gold-standard Fourier shell correlation (FSC) cut-off of 0.143(Grigorieff, 2016). The binding interface between RBD and 1F was subsequently performed Particle Subtract and Local Refinement yielding a 4.4 Å cryo-EM map showing relatively good density of the binding interface. Local resolution ranges were also analyzed within cryoSPARC.

Model building and refinement

Model building of the S trimer-1F Fab complex was performed using Coot (Emsley et al., 2010). To build the binding interface model, the 1F Fab model which was predicted using Phyre2 (Kelley et al., 2015), and one of the RBDs in the S trimer structure (PDB ID: 6VSB) were docked into the cryo-EM map of the binding interface using UCSF Chimera (Pettersen et al., 2004). The model was then manually rebuilt using Coot and real-space refined with secondary structure and geometry restraints against the map and mutation data using Phenix (Adams et al., 2010). The S trimer 1F-Fab comlex model was built using the same strategy, Models were analyzed using MolProbity (Chen et al., 2010). Cryo-EM data collection and model refinement statistics are shown in Table S5.

Structure analysis

Binding residues are defined as residues on RBD with distances to the antibody of less than 4 Å. Statistics of atom distances were performed using UCSF Chimera software (Pettersen et al., 2004). Calculations of buried surface area were performed using the same software. All the figures were generated using UCSF ChimeraX(Goddard et al., 2018), UCSF Chimera or PyMOL.

Statisitcs

The EC_{50}/IC_{50} values of ELISA, cell-cell fusion, pseudovirus neutralization, and authentic virus neutralization assays were determined using non-linear regression analysis using Graphpad PRISM. Data were presented as mean ± SD. Numbers of replicates for experiments are described in the figure legends.

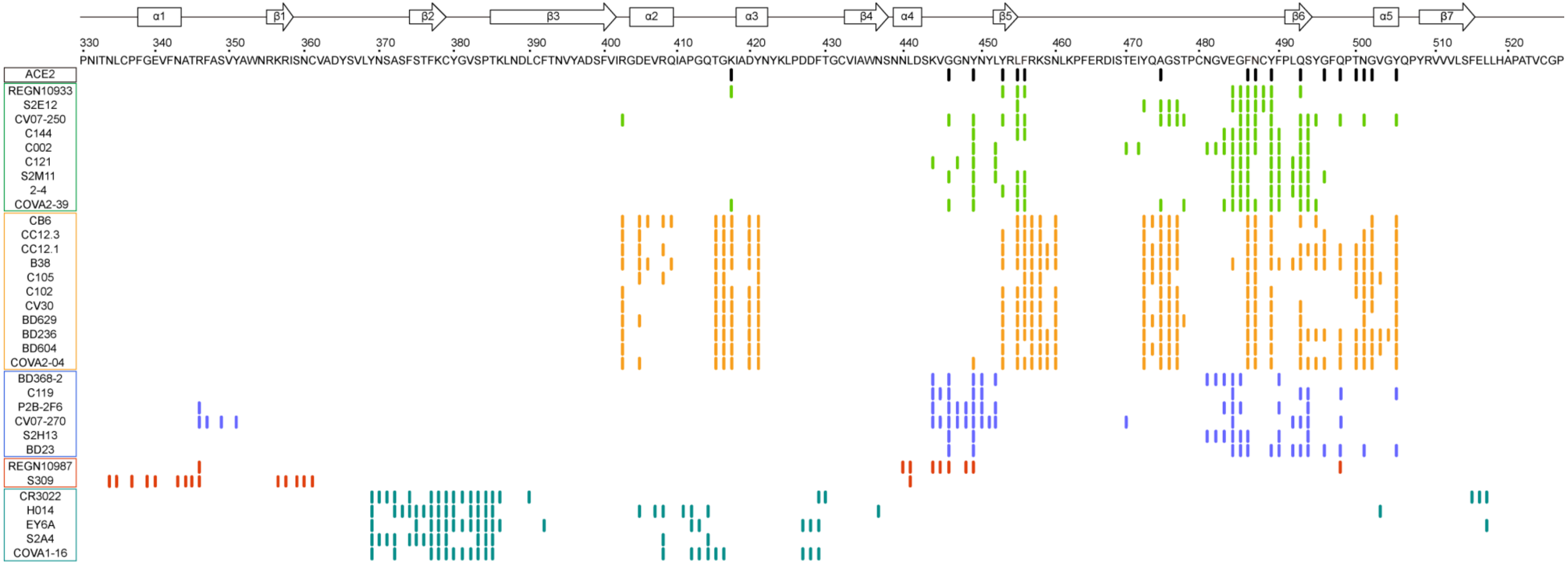

Figure S1. Interacting Residue Comparison of Structurally-resolved Antibodies against SARS-CoV-2. Related to **Figure 2A**.

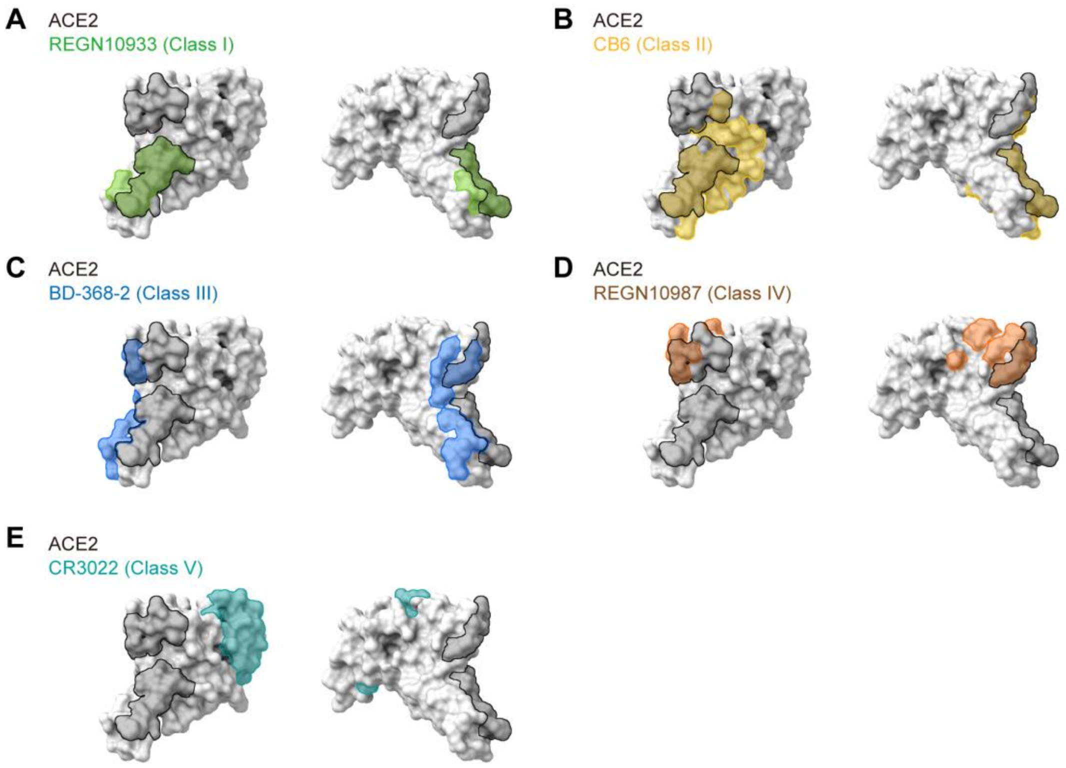

Figure S2. Epitope Comparison of Representative Antibodies in Each Class. The RBD structure is presented as surface and the interface is colored as indicated.

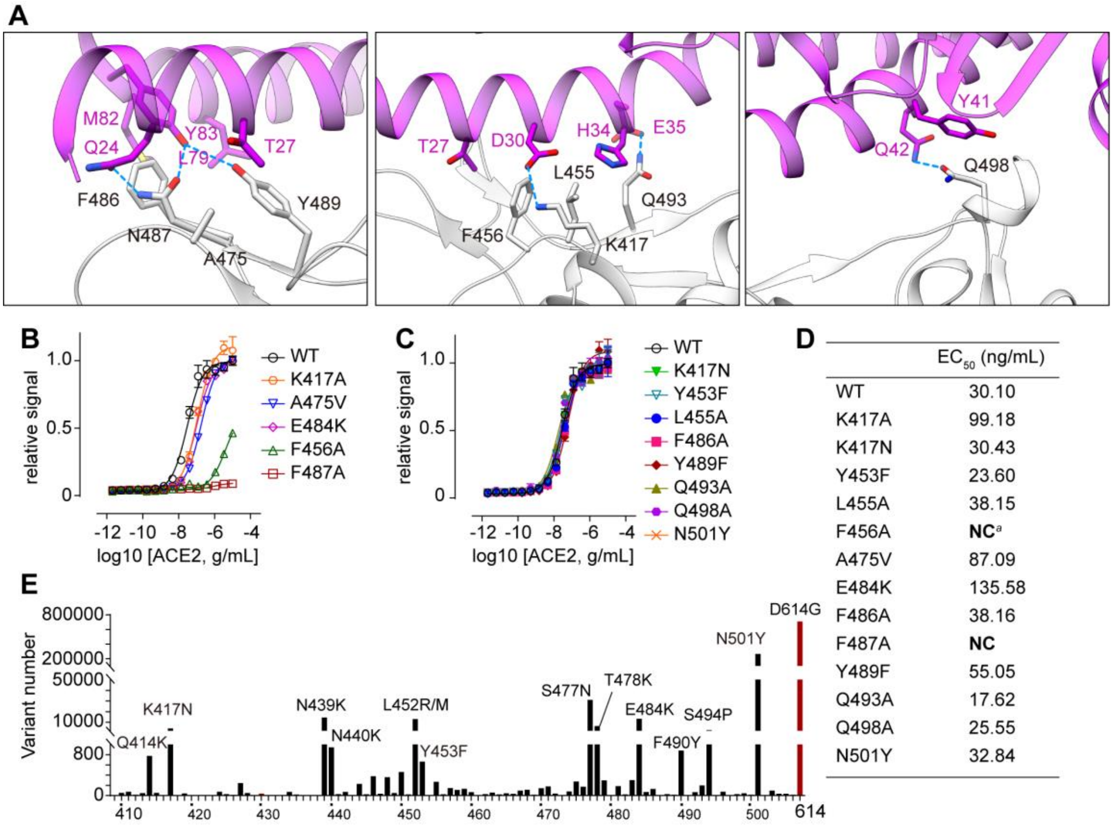

	EC_{50} (ng/mL)
WT	30.10
K417A	99.18
K417N	30.43
Y453F	23.60
L455A	38.15
F456A	**NC**[a]
A475V	87.09
E484K	135.58
F486A	38.16
F487A	**NC**
Y489F	55.05
Q493A	17.62
Q498A	25.55
N501Y	32.84

Figure S3. Key Binding Residues for Receptor Recognition. (A) Interface analysis of RBD-ACE2 complex (PDB: 6M0J), with ACE2 in pink and RBD in light gray. Binding residues selected for functional validation are highlighted as indicated. (B-D) Binding ELISA assays of mutated RBD proteins on ACE2 receptor. Experiments were performed in triplicate. (D) Summary of EC_{50} values in ELISA assays performed in (B) and (C). NC means EC_{50} cannot be calculated due to poor binding ability. (E) Summary of naturally occurred variants on RBM. Frequently occurred mutants are labelled. D614G mutant is used as reference and highlighted in red.

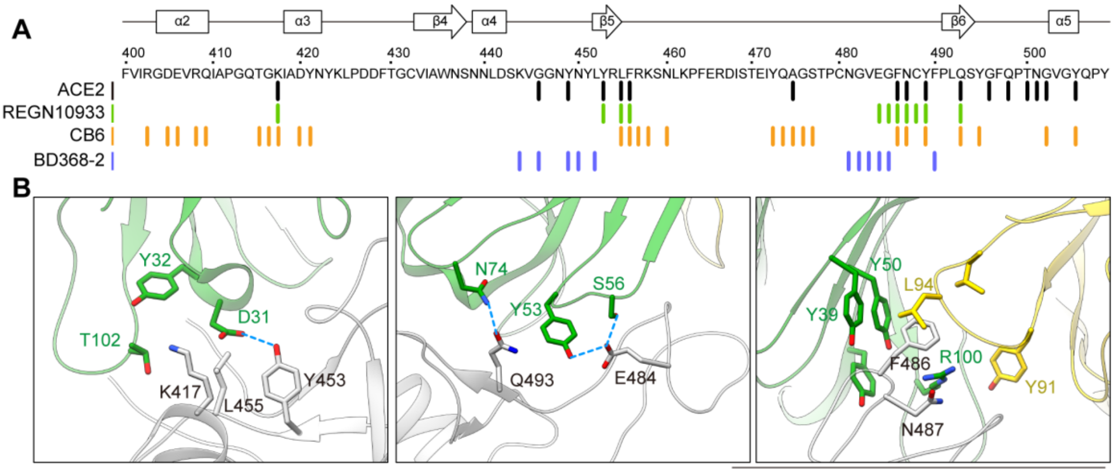

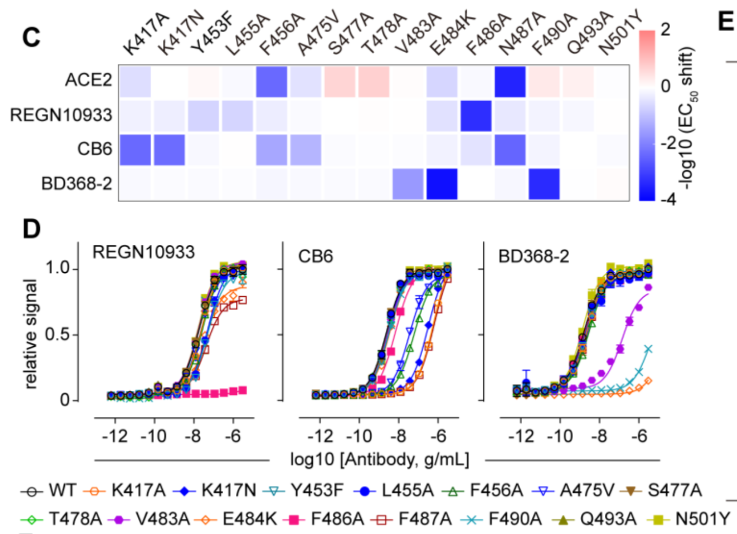

E

	EC$_{50}$ (ng/mL)		
	REGN10933	CB6	BD368-2
WT	6.48	2.40	2.80
K417A	10.98	**557.98**	3.49
K417N	11.60	**449.85**	3.40
Y453F	**31.05**	3.13	3.39
L455A	**30.14**	2.35	3.14
F456A	12.63	**58.54**	4.01
A475V	7.86	**35.36**	3.49
S477A	6.56	2.85	3.44
T478A	6.22	2.60	3.46
V483A	6.45	2.75	**113.80**
E484K	18.65	3.86	**NC**
F486A	**NC**[a]	6.82	2.86
N487A	15.01	**646.20**	3.85
F490A	10.24	3.68	**NC**
Q493A	8.96	2.43	3.43
N501Y	6.08	2.93	2.52

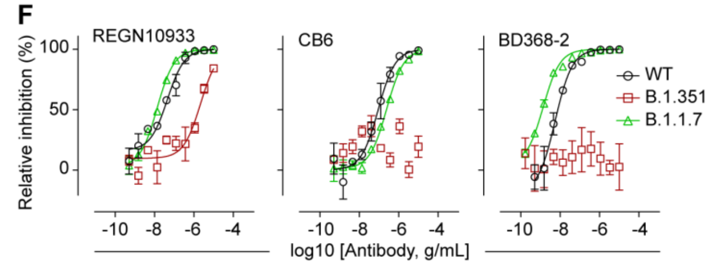

G

	IC$_{50}$ (ng/mL)		
	REGN10933	CB6	BD368-2
WT	44.60	91.56	5.59
B.1.351	**2177.0**	**NC**[a]	**NC**
B.1.1.7	13.62	246.6	1.36

Figure S4. Functional Validation of Core Epitope for SARS-CoV-2 Neutralization. (A) Binding residue comparison of ACE2 (black), and neutralizing antibodies. REGN10933 (Class I, green), CB6 (Class II, yellow), and BD368-2 (Class III, blue) are selected as representative antibodies. (B) Interface analysis of REGN10933-RBD complex (PDB: 6XDG), with the REGN-10933 Fab in green (heavy chain) and yellow (light chain). Binding residues selected for functional validation are highlighted as indicated. (C-D) Effects of mutated RBD proteins on antibody binding. (C) The -log10 (EC$_{50}$ shift) values of indicated mutants comparing to wild-type RBD in ELISA binding assay summarized as a heatmap. (D) Dose curves of mutated RBD proteins on antibody binding in ELISA assay. Experiments were performed in triplicate. (E) Summary of EC$_{50}$ values in ELISA assays performed in (D). NC means EC$_{50}$ cannot be calculated due to poor binding ability. (F-G) Neutralizing activities of REGN10933, CB6, and BD368-2 against newly emerged

variants. (F) Neutralizing Dose curves of antibodies. Experiments were performed in triplicate. (G) Summary of IC_{50} values in pseudovirus neutralization assays performed in (F).

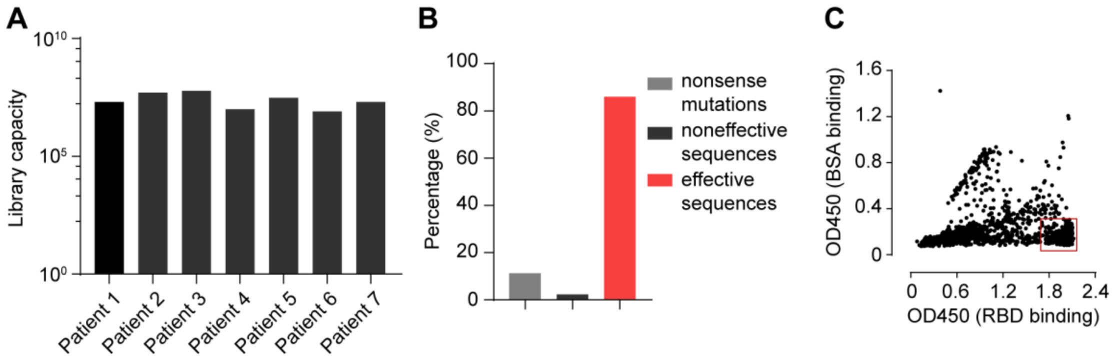

Figure S5. Quality Control of the Phage Display Libraries. (A) The capacity of the phage display libraries derived from seven patients. (B) Effective sequences quality control by sequencing. (C) Summary of single colon phage ELISA. The colons in the red box represents specific RBD binders and were selected for sequencing.

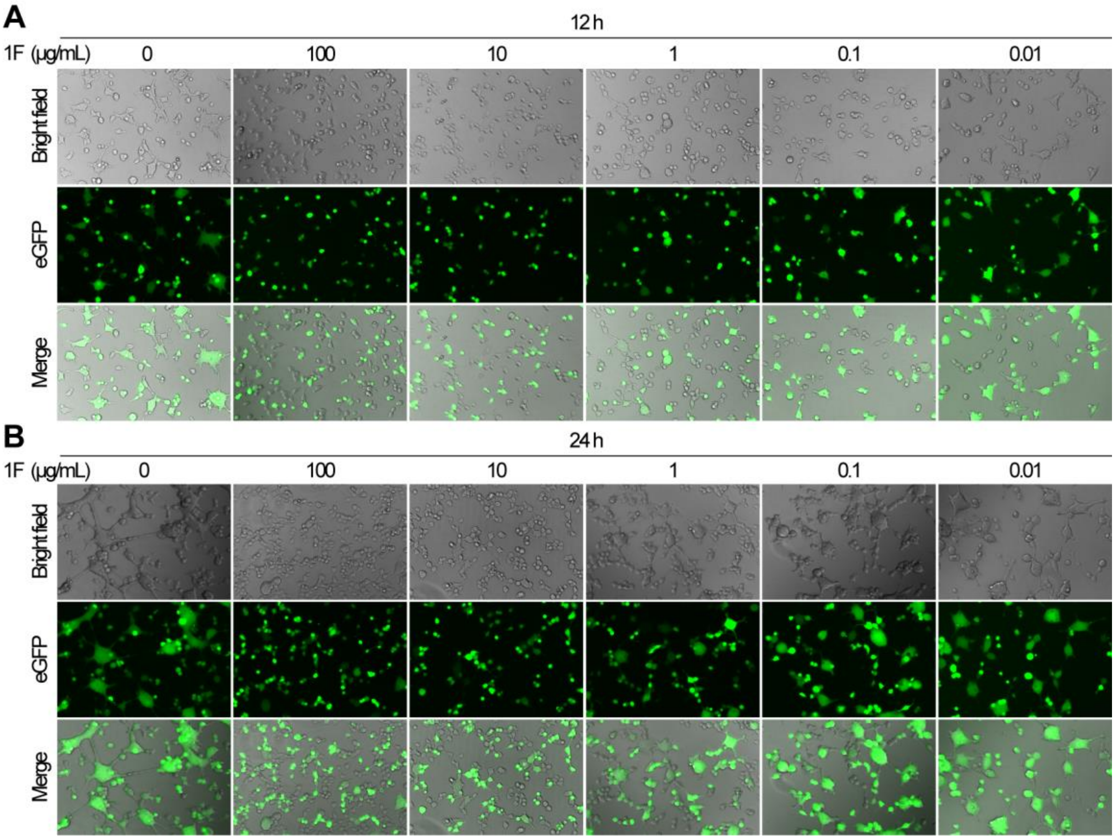

Figure S6. Representative images of SARS-CoV-2 spike mediated cell-cell fusion assay at 12 h (A) and 24 h (B).

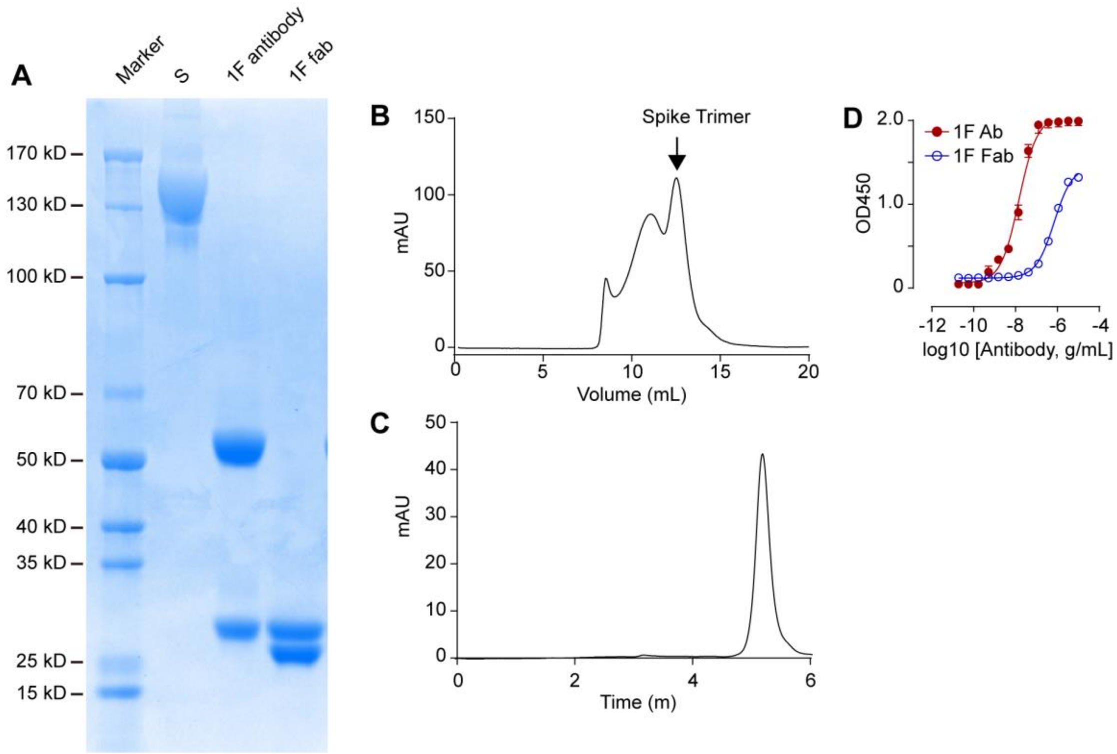

Figure S7. Purification and characterization of SARS-CoV-2 S- trimer and 1F Fab. (A) SDS-PAGE analysis of S protein, 1F IgG antibody and 1F Fab. (B) Purification of S trimer using gel filtration column. (C) SEC analysis of purified 1F Fab. (D) Binding affinities of 1F antibody and 1F fab fragment on S-trimers by ELISA. Experiments were performed in triplicates.

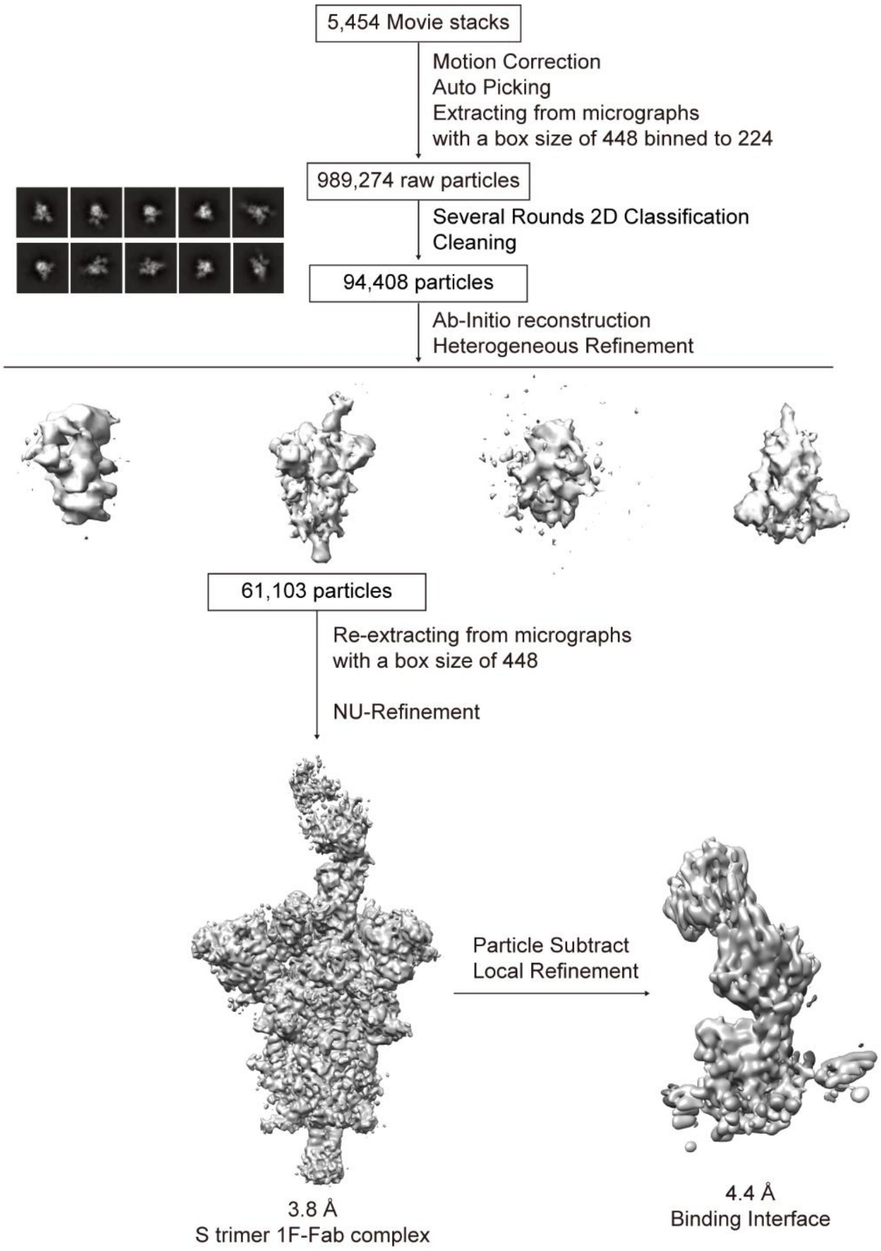

Figure S8. Flow chart for Cryo-EM data processing.

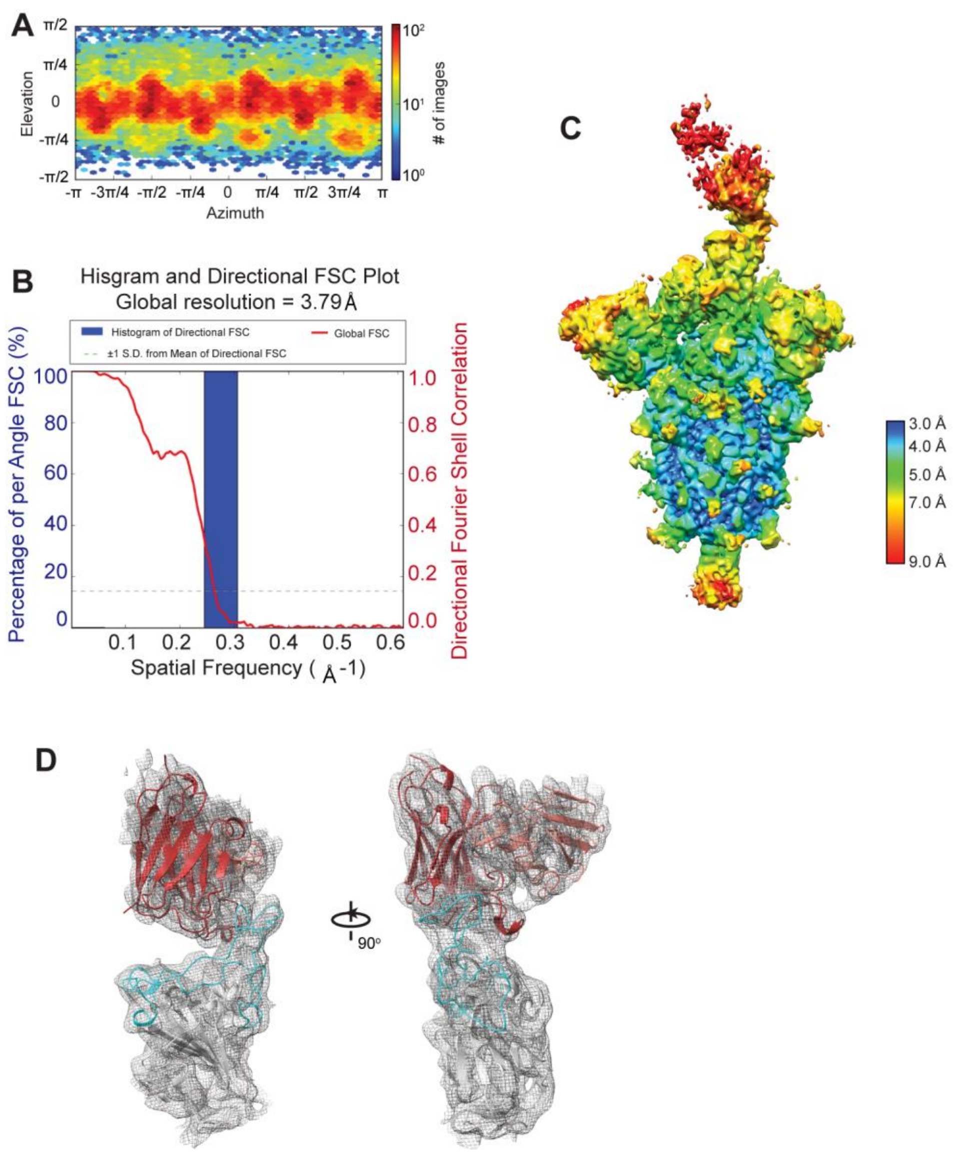

Figure S9. Cryo-EM analysis of SARS-CoV-2 S in complex with 1F Fab. (A) Cryo-EM structure view direction distribution plot. (B) The gold-standard Fourier shell correlation (FSC) curve. (C) The cryo-EM density colored according to local resolution. (D) Cartoon representation of the complex in the cryo-EM density map showing in mesh.

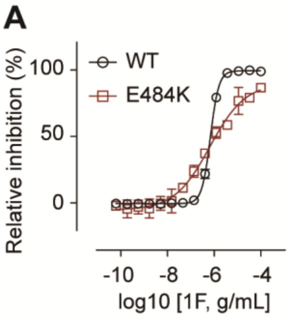

B

	IC_{50} (µg/mL)	Slope
WT	0.69	2.38
E484K	0.66	0.57

Figure S10. ACE2 binding competition of 1F on WT or E484K mutanted RBD. (A) Dose curve and (B) Summary of IC50 values and Slopes of (A). Experiments were performed in quadruplicate.

Table S1. Information Summary of Seven Convalescent Patients

Patient ID	Age (Years)	Gender	Ethnicity	The interval from disease confirmation date to blood collection date(Days)	First Symptoms
1	30	M	Chinese	31	Fever and throat discomfort
2	24	M	Chinese	39	Fever and cough
3	47	M	Chinese	45	Fever, fatigue and poor appetite
4	49	F	Chinese	31	Fever and fatigue
5	55	M	Chinese	36	Fever
6	47	F	Chinese	37	Cough and expectoration
7	49	M	Chinese	44	Fever, fatigue, and cough

Table S2. Primers Used for Immunoglobulin V_H and V_L Genes Amplification

Human Fab IgG CH1 Rv	5’- TCTTGTCCACCTTGGTGTTG -3’
VH1 Fw	5’- CAGGTCCAGCTKGTRCAGTCTGG -3’
VH157 Fw	5’- CAGGTGCAGCTGGTGSARTCTGG -3’
VH2 Fw	5’- CAGRTCACCTTGAAGGAGTCTG -3’
VH3 Fw	5’- GAGGTGCAGCTGKTGGAGWCY - 3’
VH4 Fw	5’- CAGGTGCAGCTGCAGGAGTCSG -3’
VH4 DP63 Fw	5’- CAGGTGCAGCTACAGCAGTGGG -3’
VH6 Fw	5’- CAGGTACAGCTGCAGCAGTCA -3’
scFv Fab Lambda CL1Rv	5’- TGAACATTCTGTAGGGGCCACTG -3’
scFv Fab Lambda CL2 Rv	5’- TGAACATTCCGTAGGGGCAACTG -3’
scFv Fab kappa CL Rv	5’- ACACTCTCCCCTGTTGAAGCTCTT -3’
VL 1 Fw	5’- CAGTCTGTSBTGACGCAGCCGCC -3’
VL 1459 Fw	5’- CAGCCWGKGCTGACTCAGCCMCC -3’
VL 15,910 Fw	5’- CAGTCTGYYCTGAYTCAGCCT -3’
VL 2 Fw	5’- TCCTATGWGCTGACWCAGCCAA -3’
VL 3 Fw	5’- TCCTCTGAGCTGASTCAGGASCC -3’
VL 3DPL16 Fw	5’- TCCTATGAGCTGAYRCAGCYACC -3’
VL 338 Fw	5’- AATTTTATGCTGACTCAGCCCC -3’
VK 1 Fw	5’ - GACATCCRGDTGACCCAGTCTCC -3’
VK 246 Fw	5’- GGATATTGTGMTGACBCAGWCTCC-3’
VK 3 Fw	5’ - GGAAATTGTRWTGACRCAGTCTCC-3’
VK 5 Fw	5’ - GGAAACGACACTCACGCAGTCTC-3’

Table S3. Primers Used for Introducing Flanking Homologous Arm for Gibson Assembly

Human Fab IgG CH1 Rv-2	AATCACCGGAACCAGAGGGCCGGCCgcTCTTGTCCACCTTGGTGTTG
VH1 Fw-2	CAAACGCGTACGCTGAGATCTCCCAGGTCCAGCTKGTRCAGTCTGG
VH157 Fw-2	CAAACGCGTACGCTGAGATCTCCCAGGTGCAGCTGGTGSARTCTGG
VH2 Fw-2	CAAACGCGTACGCTGAGATCTCCCAGRTCACCTTGAAGGAGTCTG
VH3 Fw-2	CAAACGCGTACGCTGAGATCTCCGAGGTGCAGCTGKTGGAGWCY
VH4 Fw-2	CAAACGCGTACGCTGAGATCTCCCAGGTGCAGCTGCAGGAGTCSG
VH4 DP63 Fw-2	CAAACGCGTACGCTGAGATCTCCCAGGTGCAGCTACAGCAGTGGG
VH6 Fw-2	CAAACGCGTACGCTGAGATCTCCCAGGTACAGCTGCAGCAGTCA
scFv Fab Lambda CL1Rv-2	GTAGTCTGCTTTGCTCAGCCTCGAGTTAATTATTTGTCATCGTCATCTTTG TAATCTGAACATTCTGTAGGGGCCACTG
scFv Fab Lambda CL2 Rv-2	GTAGTCTGCTTTGCTCAGCCTCGAGTTAATTATTTGTCATCGTCATCTTTG TAATCTGAACATTCCGTAGGGGCAACTG
scFv Fab kappa CL Rv-2	GTAGTCTGCTTTGCTCAGCCTCGAGTTAATTATTTGTCATCGTCATCTTTG TAATCACACTCTCCCCTGTTGAAGCTCTT
VL 1 Fw-2	CTATTGCTACAAATGCCTATGCATCCCAGTCTGTSBTGACGCAGCCGCC
VL 1459 Fw-2	CTATTGCTACAAATGCCTATGCATCCCAGCCWGKGCTGACTCAGCCMCC
VL 15,910 Fw-2	CTATTGCTACAAATGCCTATGCATCCCAGTCTGYYCTGAYTCAGCCT
VL 2 Fw-2	CTATTGCTACAAATGCCTATGCATCCTCCTATGWGCTGACWCAGCCAA
VL 3 Fw-2	CTATTGCTACAAATGCCTATGCATCCTCCTCTGAGCTGASTCAGGASCC
VL 3DPL16 Fw-2	CTATTGCTACAAATGCCTATGCATCCTCCTATGAGCTGAYRCAGCYACC
VL 338 Fw-2	CTATTGCTACAAATGCCTATGCATCCAATTTTATGCTGACTCAGCCCC
VK 1 Fw-2	CTATTGCTACAAATGCCTATGCATCCGACATCCRGDTGACCCAGTCTCC
VK 246 Fw-2	CTATTGCTACAAATGCCTATGCATCCGGATATTGTGMTGACBCAGWCTCC
VK 3 Fw-2	CTATTGCTACAAATGCCTATGCATCCGGAAATTGTRWTGACRCAGTCTCC
VK 5 Fw-2	CTATTGCTACAAATGCCTATGCATCCGGAAACGACACTCACGCAGTCTC

Table S4. Heavy and light chain sequences of 1F

Heavy Chain[a]	EVQLVESGPGLVKPSETLSLTCTVSGGSISSSSYYWGWIRQPPGKGL EWIGSIYYRGSTYYNPSLKSRVTISVDTSKNQFSLKLSSVTAADTAV YYCARHVRSAYYYGSGSYRDEGNWFDPWGQGTLVTVSS
Light Chain	QSVLTQPPSVSGAPGQRVTISCTGTRSNIGAGHDVHWYQQLPGTAP KLLIYGNNNRPSGVPDRFSGAKSGTSASLAITGLQAEDEADYYCQS YDRTLTSYVFGTGTKVTVL

[a] CDRs were highlighted in red

Table S5. Summary of the Cyro-EM structure

	S trimer 1F-Fab complex	Binding interface
PDB ID	7Y7J	7Y7K
EMDB ID	EMD-33667	EMD-33668
Microscope	FEI Titan Krios	FEI Titan Krios
Magnification	29000	29000
Voltage (kV)	300	300
Electron exposure ($e^-/Å^2$)	60	60
Defocus range (μm)	-1.0 to -2.5	-1.0 to -2.5
Pixel size (Å)	0.82	0.82
Number of movies	5,454	5,454
Symmetry imposed	C1	C1
Final particle images (no.)	61,103	61,103
Map resolution (Å)<br>FSC threshold	3.8<br>0.143	4.4<br>0.143
**Refinement**		
Map sharpening *B* factor ($Å^2$)	-84.3	-100.4
Model composition<br>Non-hydrogen atoms<br>Protein residues<br>Ligands	 23,798<br>3,191<br>60	 2,942<br>422
R.m.s. deviations<br>Bond lengths (Å)<br>Bond angles (°)	 0.007<br>0.962	 0.010<br>1.628
Validation<br>Clashscore<br>Poor rotamers (%)	 11.99<br>1.09	 33.39<br>3.90
Ramachandran plot<br>Favored (%)<br>Allowed (%)<br>Disallowed (%)	 93.66<br>5.56<br>0.77	 86.06<br>10.34<br>3.61

Reference

Adams, P.D., Afonine, P.V., Bunkoczi, G., Chen, V.B., Davis, I.W., Echols, N., Headd, J.J., Hung, L.W., Kapral, G.J., Grosse-Kunstleve, R.W*., et al.* (2010). PHENIX: a comprehensive Python-based system for macromolecular structure solution. Acta Crystallogr D Biol Crystallogr *66*, 213-221.

Chen, V.B., Arendall, W.B., 3rd, Headd, J.J., Keedy, D.A., Immormino, R.M., Kapral, G.J., Murray, L.W., Richardson, J.S., and Richardson, D.C. (2010). MolProbity: all-atom structure validation for macromolecular crystallography. Acta Crystallogr D Biol Crystallogr *66*, 12-21.

Emsley, P., Lohkamp, B., Scott, W.G., and Cowtan, K. (2010). Features and development of Coot. Acta Crystallogr D Biol Crystallogr *66*, 486-501.

Goddard, T.D., Huang, C.C., Meng, E.C., Pettersen, E.F., Couch, G.S., Morris, J.H., and Ferrin, T.E. (2018). UCSF ChimeraX: Meeting modern challenges in visualization and analysis. Protein Sci *27*, 14-25.

Grigorieff, N. (2016). Frealign: An Exploratory Tool for Single-Particle Cryo-EM. Methods Enzymol *579*, 191-226.

Kelley, L.A., Mezulis, S., Yates, C.M., Wass, M.N., and Sternberg, M.J.E. (2015). The Phyre2 web portal for protein modeling, prediction and analysis. Nature Protocols *10*, 845-858.

Mastronarde, D.N. (2005). Automated electron microscope tomography using robust prediction of specimen movements. J Struct Biol *152*, 36-51.

Pettersen, E.F., Goddard, T.D., Huang, C.C., Couch, G.S., Greenblatt, D.M., Meng, E.C., and Ferrin, T.E. (2004). UCSF Chimera--a visualization system for exploratory research and analysis. J Comput Chem *25*, 1605-1612.

Punjani, A., Rubinstein, J.L., Fleet, D.J., and Brubaker, M.A. (2017). cryoSPARC: algorithms for rapid unsupervised cryo-EM structure determination. Nat Methods *14*, 290-296.

Tonikian, R., Zhang, Y., Boone, C., and Sidhu, S.S. (2007). Identifying specificity profiles for peptide recognition modules from phage-displayed peptide libraries. Nat Protoc *2*, 1368-1386.

Wrapp, D., Wang, N., Corbett, K.S., Goldsmith, J.A., Hsieh, C.-L., Abiona, O., Graham, B.S., and McLellan, J.S. (2020). Cryo-EM structure of the 2019-nCoV spike in the prefusion conformation. Science *367*, 1260.

Xia, S., Liu, M., Wang, C., Xu, W., Lan, Q., Feng, S., Qi, F., Bao, L., Du, L., Liu, S*., et al.* (2020). Inhibition of SARS-CoV-2 (previously 2019-nCoV) infection by a highly potent pan-coronavirus fusion inhibitor targeting its spike protein that harbors a high capacity to mediate membrane fusion. Cell Res *30*, 343-355.

Zheng, S.Q., Palovcak, E., Armache, J.P., Verba, K.A., Cheng, Y., and Agard, D.A. (2017). MotionCor2: anisotropic correction of beam-induced motion for improved cryo-electron microscopy. Nat Methods *14*, 331-332.
